# Ischemic Colitis or Colon Cancer: A Diagnostic Dilemma

**DOI:** 10.7759/cureus.36206

**Published:** 2023-03-15

**Authors:** Sneha Khanal, Tanushree Bhatt, Patrik Schmidt, Priscilla L Hallal, May Zaw

**Affiliations:** 1 Internal Medicine, BronxCare Health System, The Bronx, USA

**Keywords:** colon mass, colonoscopy, ischemic colitis, colon carcinoma, mass-forming ischemic colitis

## Abstract

Ischemic colitis, a potentially reversible pathology of the colon, can masquerade in its presentation as colonic carcinoma. It typically presents with cramping abdominal pain, diarrhea, and per-rectal bleeding. Colonoscopy remains the diagnostic modality of choice that typically shows friable, edematous, or erythematous mucosa with scattered hemorrhagic erosions or ulcerations. Although rare, the colonoscopic findings can sometimes reveal a tumor mass that confounds the diagnosis of ischemic colitis as colonic carcinoma. Our patient was a 78-year-old female with no previous colon cancer screening who presented with a mass-forming variant of ischemic colitis. Due to the overlap in presentations, radiographic findings, and colonoscopic findings, the diagnostic challenge was evident. Ultimately, colon cancer was ruled out through thorough colonoscopic follow-up and biopsy-guided pathological analysis. This case signifies the importance of considering colonic mass as a guise of underlying ischemic colitis to ensure an accurate diagnosis and the best possible outcome for the patient.

## Introduction

Ischemic colitis is a gastrointestinal vascular pathology characterized by a diminished or complete lack of blood supply to the colon, causing an acute insult. Its estimated incidence in the general population is 4.5-44/100,000 years [1]. Ischemic colitis has been classified as non-occlusive and occlusive types. The non-occlusive variant, which is the most common type, is caused by conditions resulting in low blood flow to the colon, namely hypotension, dehydration, drugs, constipation, coagulopathy, septic shock, and surgeries. On the other hand, the occlusive type is caused by conditions that result in occlusions of the colonic vasculature, such as neoplasms, thrombosis, emboli, volvulus, and adhesions [2]. 

Ischemic colitis has multiple ways of presenting itself; the clinical diagnosis is aided by radiography, colonoscopy, biopsy, and cytopathological examinations. One of the biggest challenges in the diagnosis of ischemic colitis is its rare mass-forming presentation that can be easily confused with colon cancer. This atypical presentation of ischemic colitis has been described in the present case. 

## Case presentation

A 78-year-old female presented to the emergency department with cramping right lower quadrant abdominal pain for two days. The pain was gradual in onset, four out of 10 in intensity, non-radiating, and had no aggravating or relieving factors. The abdominal pain was associated with multiple episodes of diarrhea and bright red bleeding per rectum. The patient did not report unintentional weight loss and did not have a history of smoking, alcohol use, illicit drug use, or allergies. Medical history included hypertension, hyperlipidemia, and hypothyroidism for more than 15 years. She was under lisinopril and amlodipine for hypertension, which was well-controlled. She was under levothyroxine for maintenance of her thyroid status and was recently diagnosed with pre-diabetes, which was under control with diet, exercise, and lifestyle modifications. She did not take any over-the-counter or herbal medications. She was under atorvastatin for hyperlipidemia. Her family history was significant for pancreatic cancer in her mother.

**Figure 1:**
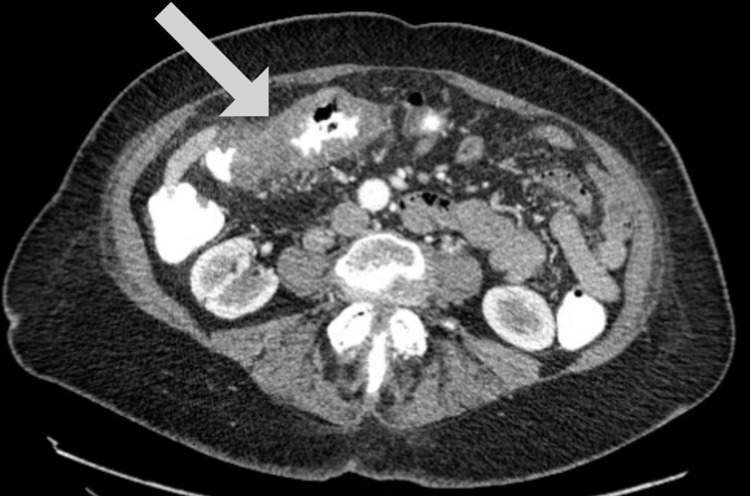
CT abdomen and pelvis showing mural thickening of transverse colon at the hepatic flexure with narrow lumen as demarcated by the arrow.

On presentation, the patient had stable vitals. Physical examination showed right lower quadrant abdominal tenderness and bright red blood on the finger on rectal examination. Laboratory tests revealed leukocytosis, hypernatremia, and hypokalemia. Other laboratory investigations were within the reference range, including hemoglobin, blood urea nitrogen, and serum creatinine (Table 1). Computed tomography (CT) of the abdomen and pelvis with contrast demonstrated mural thickening of the transverse colon at the hepatic flexure with significant luminal narrowing that was initially suspected to be due to colitis (Figure 1).

**Table 1:** Laboratory parameters upon admission

Laboratory parameters	Values	Reference ranges
White blood cells	14.7 K/uL	4.8-10.8 K/uL
Hemoglobin	13.3 gm/dl	12-16 gm/dl
Hematocrit	39.8%	42-51%
Platelets	233 K/uL	150-400 K/uL
Sodium	147 mEq/L	135-145 mEq/L
Potassium	2.9 mEq/L	3.5-5.0 mEq/L
Blood urea nitrogen	10 mg/dl	6-20 mg/dl
Creatinine	0.7 mg/dl	0.5-1.5 mg/dl
Stool for occult blood	Positive	Negative

**Figure 2:**
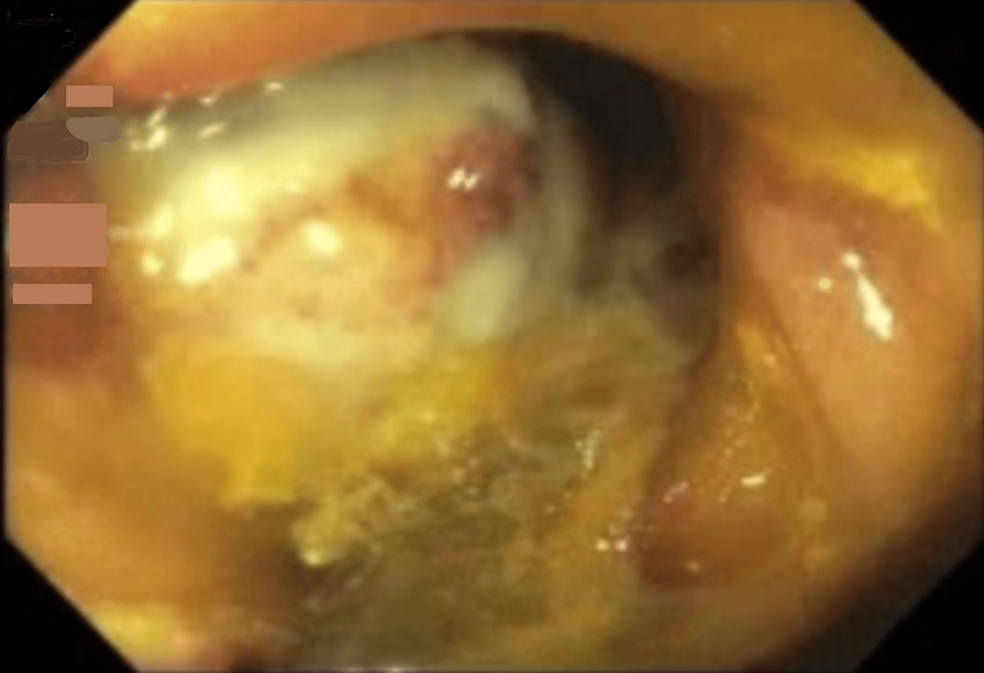
Large, non-obstructing polypoid mass with overlying ulceration in the transverse colon on initial colonoscopy.

**Figure 3:**
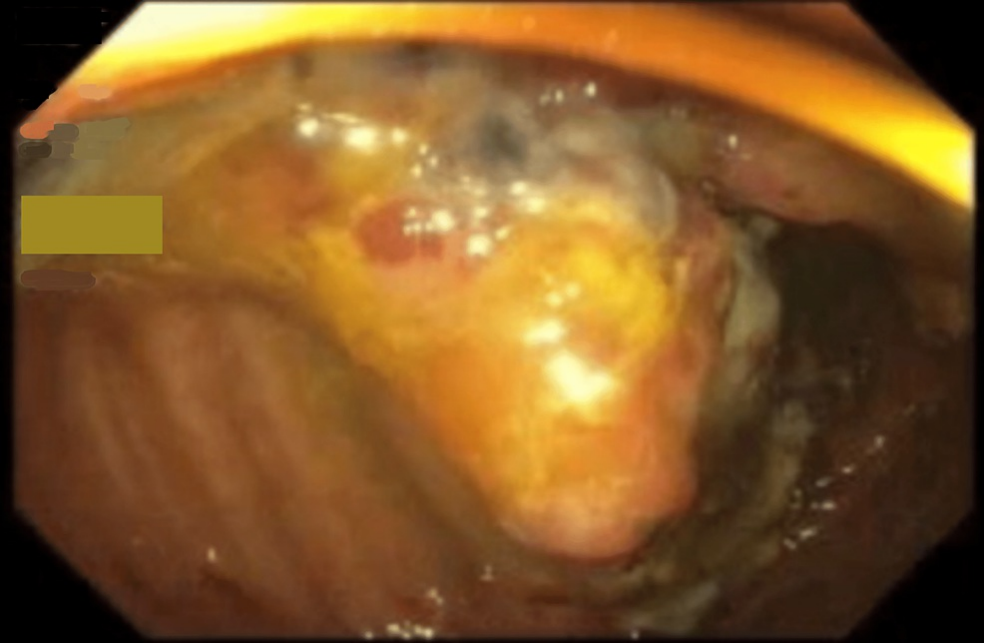
Large, non-obstructing polypoid mass with overlying ulceration in the transverse colon on initial colonoscopy.

The patient was treated for suspected colitis with intravenous fluids, antibiotics, and bowel rest. A colonoscopy revealed a polypoid and ulcerated non-obstructing large mass, 10 cm in length in the transverse colon concerning for colon cancer (Figure 2-3). No bleeding was found. The pathological analyses from the biopsies reported hyperplastic changes in the colonic mucosa, focal ulceration, extravasated red blood cells, and glandular atrophy suggestive of ischemic changes. No evidence of malignancy was evident in the biopsies. Carcinoembryonic antigen (CA) level was 3.8 ng/ml (reference range: <5 ng/ml), and CA 19-9 was 2.1 u/ml (reference range: 0-37 u/ml).

**Figure 4:**
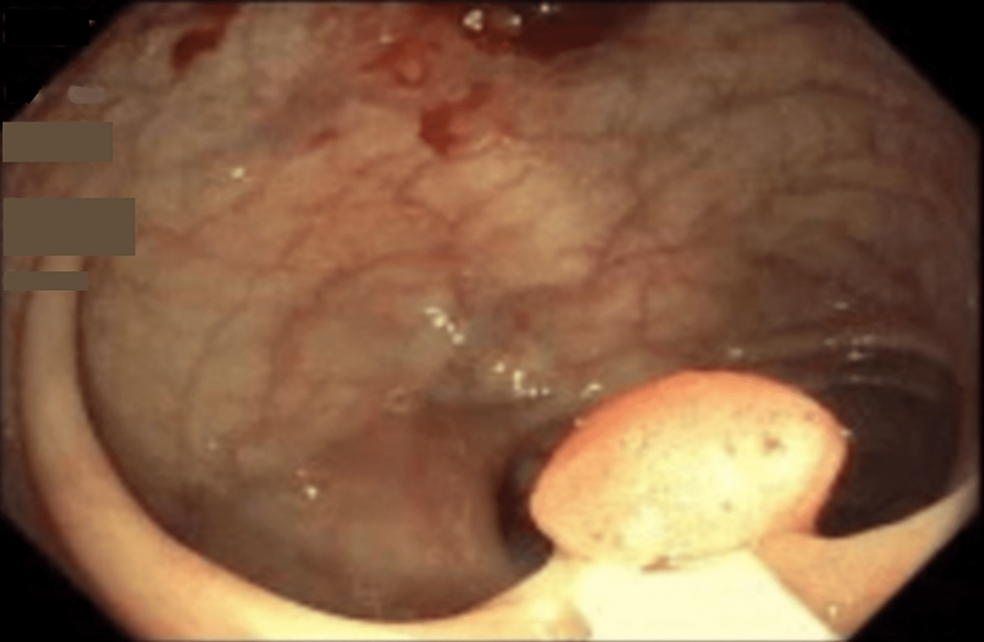
Complete resolution of the previously seen colonic mass upon repeat colonoscopy. A small sessile polyp noted in the splenic flexure.

**Figure 5:**
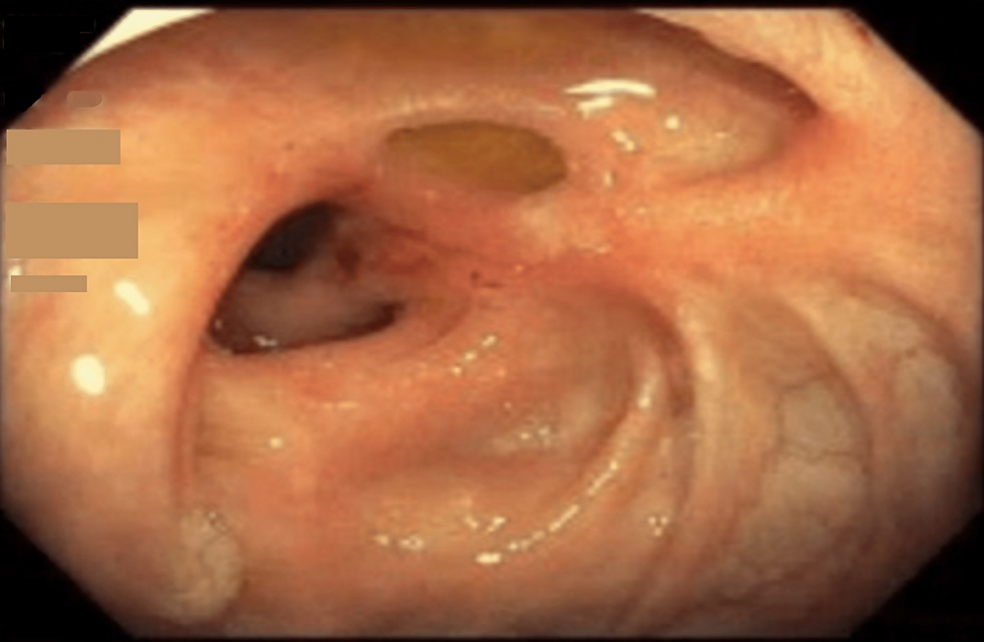
Complete resolution of the previously seen colonic mass upon repeat colonoscopy.

The patient was discharged with conservative management. The patient completed seven days of antimicrobial therapy with metronidazole and ceftriaxone inpatient and metronidazole and ciprofloxacin on discharge and had bowel rest with the gradual resumption of diet. The patient was also educated to avoid dehydration, low blood pressure, and constipation. A colonoscopy repeated after two months revealed a 12 mm sessile polyp in the splenic flexure and a 3 mm sessile polyp in the cecum (Figure 4). The previously visualized colonic mass was not seen, and there was an interval healing of the ischemic colitis (Figure 5). The patient reported complete resolution of her abdominal symptoms and had no further episodes of pain or bleeding. 

## Discussion

The large intestines have a rich vascular supply. The branches of the superior mesenteric artery (SMA), inferior mesenteric artery (IMA), and the communication between SMA and IMA that run parallel throughout the colon as the marginal artery create an intricate network for colonic perfusion ​[1]. Despite this extensive blood supply, the colon is prone to ischemic insults, particularly secondary to splanchnic vasoconstriction or due to increased intra-luminal pressure causing diminished micro-vascularization of the colonic wall ​[2]. Multiple etiologies contribute to the pathology of ischemic colitis, such as compromised blood flow in systemic circulation as well as obstructions due to alterations of anatomic or physiological homeostasis in the colon ​[3]. The various causes of ischemic colitis are given in Table 2 [4].

**Table 2:** Causes of ischemic colitis

Causes of ischemic colitis	
Colonic vasculature occlusion	Mesenteric arterial thrombosis, emboli
Colonic obstruction	Neoplasms, adhesions, chronic constipation, intestinal prolapse, volvulus
Low flow states	Shock, anaphylaxis, heart failure, trauma
Hypercoagulable states	Protein C deficiency, protein S deficiency, antithrombin deficiency, anti-phospholipid antibody syndrome
Intra-abdominal infections	*Escherichia coli*, *Shigella*, *Salmonella*, cytomegalovirus,* Campylobacter enterocolitis *
Vasculitis	Systemic lupus erythematosus, polyarteritis nodosa, rheumatoid arthritis, granulomatosis with polyangiitis
Surgeries	Aortic dissection repair, aortic aneurysm repair, coronary artery bypass surgery
Medications/drugs	Cocaine, amphetamine, diuretics, nonsteroidal anti-inflammatory drugs, digoxin, estrogen, oral contraceptives, vasopressin, sumatriptan, antihypertensives, pseudoephedrine

The diagnosis of ischemic colitis is usually clinical. The patients typically present with cramping abdominal pain, diarrhea, and per-rectal bleeding. However, varied presentations of the condition ranging from abdominal discomfort to acute abdomen can render the diagnosis of ischemic colitis to be challenging at times [5]. Other conditions associated with rectal bleeding, such as polyps, cancer, diverticulitis, inflammatory diseases, and vascular malformations, may confound the diagnosis [6]. Imaging modalities and colonoscopy with biopsies and cytopathology can aid as an ancillary tool in confirming the diagnosis. The gastrointestinal insult caused by ischemic colitis can range from superficial inflammation to full-thickness involvement of the colon leading to gangrene formation. A colonoscopy typically shows friable, edematous, or erythematous mucosa with scattered hemorrhagic erosions or ulcerations [7,8]. Nonetheless, sometimes they may present as strictures of the colonic segment, deep-seated ulcerations, and rarely a façade of a tumor mass [9]. 

At least 20% of cases of colonic ischemia have been attributed to a benign or malignant colonic mass [10,11]. The area of ischemia in such cases is either proximal to or within the space occupied by the mass. The differentiation of the mass-forming variant of ischemic colitis from an obstructive colonic mass causing secondary ischemia to the colon poses a diagnostic challenge as there is a significant overlap in the clinical, imaging, and colonoscopic findings [12]. Often this predicament warrants premature surgical consultation and causes a psychosocial burden upon the patients and their families. 

Our patient had multiple cardiovascular risk factors, that might have contributed to the development of ischemic colitis. Although she did not have a history of illicit substance use or smoking, and no history of overt hypotension or severe constipation, the patient might have developed atheromatous plaque contributing to the development of ischemic colitis. Ischemic colitis in our patient could have also resulted from intermittent chronic constipation, which was unaccounted for during the initial presentation due to its mild nature, which is the limitation of our case. However, other pathologies including neoplasms, emboli, volvulus, adhesions, and coagulopathies were ruled out. The patient was educated about the avoidance of constipation, adequate control of her cardiovascular risk factors, and the significance of regular follow-up to prevent the recurrence of her condition.

Most cases of ischemic colitis are successfully treated medically with bowel rest, intravenous fluids, electrolyte replenishment, and broad-spectrum antibiotics. The treatment of underlying etiology remains an important facet of its management. Surgical intervention in the form of extended colectomy may be indicated in case of severe complications such as peritonitis. Surgical management, when necessary, is associated with significantly high mortality rates (39.3%) as compared to medical management (6.2%) [5]. In rare cases of mass-forming ischemic colitis, if the symptoms are not severe and pathology is consistent only with inflammation, a colonoscopy should be repeated before proceeding with any planned surgical intervention to avoid serious complications. 

## Conclusions

The life-threatening colonic carcinoma needs to be vigilantly ruled out, albeit this mass-forming presentation of ischemic colitis. Reports of coexisting colonic carcinoma and ischemic colitis remain abundant; however, vanishing colonic masses like those described here are atypical and captivating clinical presentations of ischemic colitis. The vague clinical presentation, non-specific radiological findings, and the imitative mass seen on direct visualization all contribute to the struggle in differentiating the two. Our case report aims to highlight early recognition of such unusual presentations of ischemic colitis to avoid needless surgeries and associated complications including adhesions, strictures, and even an increase in mortality. 
